# Supplementary material for: ConfuseNN: Interpreting convolutional neural network inferences in population genomics with data shuffling
Source: bioRxiv. 2025 Mar 27:2025.03.24.644668. Preprint. [Version 1] doi: 10.1101/2025.03.24.644668 (PMC11974698; doi:10.1101/2025.03.24.644668)
Supplement: Supplement 1 [file NIHPP2025.03.24.644668v1-supplement-1.pdf]

bioRxiv preprint doi: https://doi.org/10.1101/2025.03.24.644668; this version posted March 27, 2025. The copyright holder for this preprint (which was not certified by peer review) is the author/funder, who has granted bioRxiv a license to display the preprint in perpetuity. It is made available under aCC-BY 4.0 International license.

Supplementary Methods

Positive selection classifier CNN - disc-pg-gan

The original study trained several discriminators using different human populations and seed. We analyzed the behavior of a discriminator CNN trained on CEU population data seed 19 from the original publication. This CNN has been fine-tuned with positive selection simulations generated using SLiM.

To generate simulated test data, we used the Riley et al. (2024) SLiM-based procedure to generate 3000 simulated genomic regions for the neutral case (true negative in the binary test) and 2400 simulated genomic regions for positive selection case (true positive), using four positive selection strengths (selection coefficient $s = 0.01, 0.025, 0.05, 0.1$), each contributing 600 simulated genomic regions.

Positive selection classifier CNN - Imagene

Training and test simulations were generated with *msms* using a three-epoch demographic history model with specified parameters from (Marth et al. 2004) for the CEU population, following Torada et al. (2019). The simulated genomic regions were of size 80 kb and 128 haplotypes were sampled from CEU individuals. The demographic history model has two instantaneous population size change events: a bottleneck of $10,000$ down to $2,000$ individuals at 3500 generations before the present and a recovery and expansion to $20,000$ at 3000 generations before the present. To incorporate selective sweeps, 5000 simulations were generated per selection coefficients $S = 0, 200, 400$ in $2N$ units, which correspond to $S = 0, 0.01, 0.02$.

Each simulation was processed into a 128×128 haplotype matrix using major/minor allele polarization and removal of loci with less than 1% allele frequency. The 128 rows in the final matrix correspond to the 128 sampled haplotypes, while the variable number of segregating sites are standardized into 128 with the image resizing python package *scikit-image* (Van der Walt et al. 2014). Both rows and columns of the haplotypes were sorted by frequency of occurrence.

The network used here was implemented using python packages TensorFlow v2.15.0 and Keras v2.15.0. The network architecture consists of three convolutional layers each with 32, 64, and

bioRxiv preprint doi: https://doi.org/10.1101/2025.03.24.644668; this version posted March 27, 2025. The copyright holder for this preprint (which was not certified by peer review) is the author/funder, who has granted bioRxiv a license to display the preprint in perpetuity. It is made available under aCC-BY 4.0 International license.

64 filters respectively with a ReLU activation function followed by a max pooling layer. The output is flattened before being passed into the final output dense layer with 3 nodes corresponding to the 3 classes of selection (0, 200, and 400) and a softmax activation function.

Demographic history inference CNN

Training and test simulations were generated with *ms* (Hudson 2002) using a three-epoch demographic history model as described in Flagel et al. (2019). Briefly, 100,000 1.5MB regions were simulated with human mutation and recombination rates and demographic parameters randomly sampled from ranges described in Table S1. As done in the original study, we also divided the mutation rate by 10, which is equivalent to randomly downsampling the total number of SNPs by a factor of 10. 80,000 simulations were used in training and 20,000 simulations were used for accuracy testing as well as the scramble test.

Each simulation was processed into a haplotype matrix with 50 rows, corresponding to the number of sampled haplotypes. The width of the matrix equaled the maximum number of segregating sites observed across all simulations. For simulations with fewer sites, columns of 0s were added to the right edges (zero-padding) to make all matrices have the same fixed width.

The position vector has length equal to the maximum number of segregating sites as above minus one. Each value in this vector is the normalized distance between two adjacent segregating sites, with 1 being the total length of the 1.5 MB simulated region. Position vectors are also zero-padded so they all have the same length.

The network used here was implemented using python packages TensorFlow v1.1.0 and Keras v2.0.6. This network has two branches, one takes the haplotype matrix as input and the other takes the position vector as input. The haplotype matrix branch consists of four convolutional layers each with 128 filters followed by a max pooling layer. The position vector branch consists of a dense layer with 32 nodes. The output of these two branches are concatenated before being fed into the final dense layer of 256 nodes. We implemented the best performing hyperparameters reported in the original study, which were 1D convolution type, kernel size 2, output in log scale, drop-out layers throughout, and sorting of chromosomes by genetic similarity.

bioRxiv preprint doi: https://doi.org/10.1101/2025.03.24.644668; this version posted March 27, 2025. The copyright holder for this preprint (which was not certified by peer review) is the author/funder, who has granted bioRxiv a license to display the preprint in perpetuity. It is made available under aCC-BY 4.0 International license.

Supplementary Results

Table S1: Our accuracy scores compare to the original work in Flagel et al. (2019) when replicating the demographic history inference CNN.

	Flagel et al.	Our replication	Flagel et al.	Our replication
Parameter (range)	RMSE	RMSE	Spearman's ρ	Spearman's ρ
$N_0(100, 40000)$	N.A.	0.584	0.59	0.591
$N_1(100, 5000)$	N.A.	0.764	0.54	0.544
$N_2(100, 20000)$	N.A.	0.571	0.84	0.856
$T_1(100, 3499.99)$	N.A.	0.741	0.62	0.605
$T_2(T_1 + 1, T_1 + 3500)$	N.A.	0.674	0.62	0.609
RMSE on validation set	0.43366	0.451		

bioRxiv preprint doi: https://doi.org/10.1101/2025.03.24.644668; this version posted March 27, 2025. The copyright holder for this preprint (which was not certified by peer review) is the author/funder, who has granted bioRxiv a license to display the preprint in perpetuity. It is made available under aCC-BY 4.0 International license.

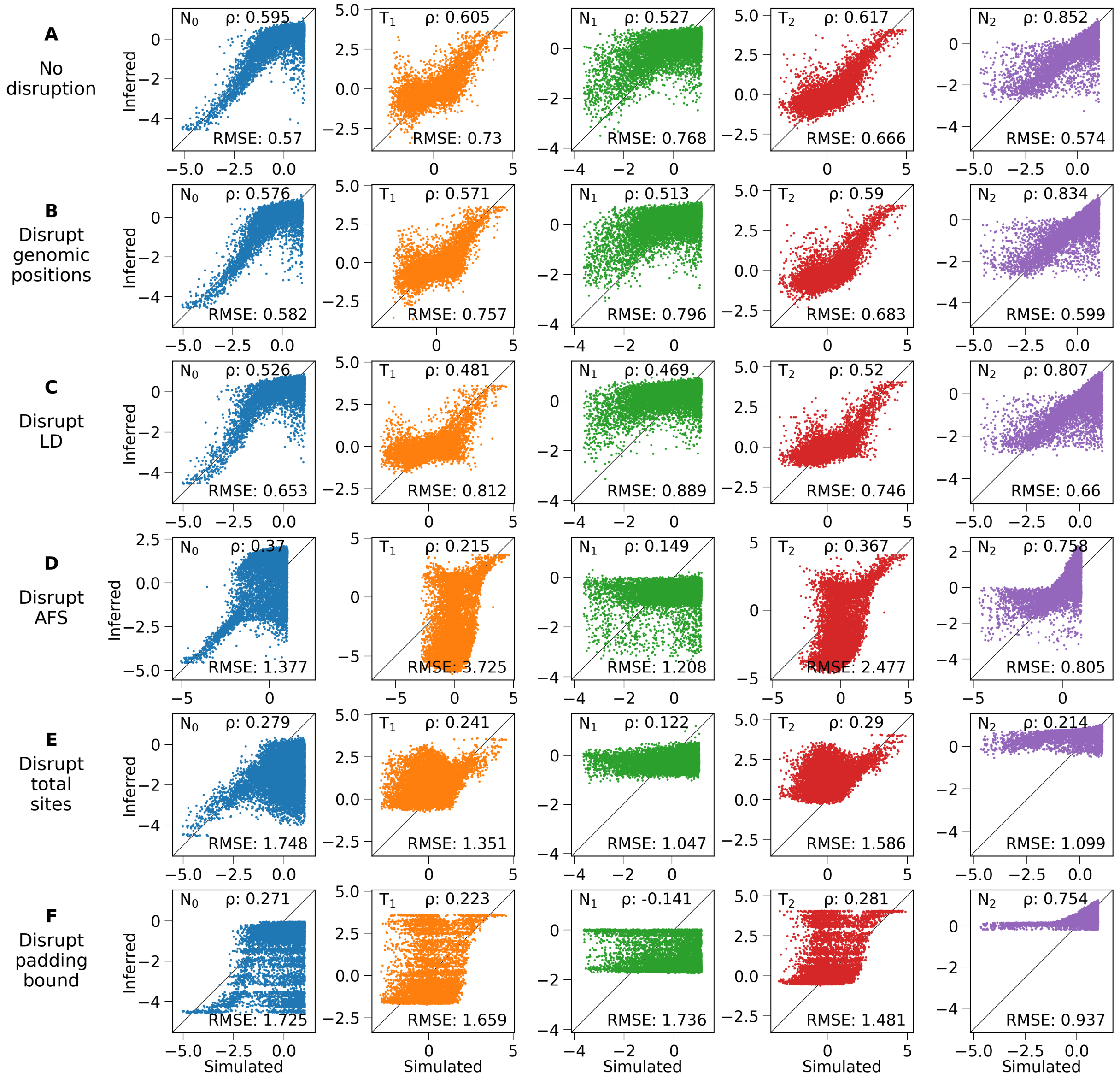

Figure S1: **Effects of data shuffling on Flagel et al.'s demographic history inference CNN.** (A) Replicating the original CNN's performance before shuffling. (B-F) The accuracy of the original CNN when tested on different shuffled test data shown in Fig. 1. Accuracy is measured by the Spearman's rank coefficient ρ and the root mean squared error (RMSE) as per the original work. All parameter values are in log scale.

REFERENCES

Flagel L, Brandvain Y, Schrider DR (2019) The unreasonable effectiveness of convolutional neural networks in population genetic inference. Molecular biology and evolution 36:220.

bioRxiv preprint doi: https://doi.org/10.1101/2025.03.24.644668; this version posted March 27, 2025. The copyright holder for this preprint (which was not certified by peer review) is the author/funder, who has granted bioRxiv a license to display the preprint in perpetuity. It is made available under aCC-BY 4.0 International license.

Hudson RR (2002) Generating samples under a wright–fisher neutral model of genetic variation. Bioinformatics 18:337.

Marth GT, Czabarka E, Murvai J, Sherry ST (2004) The allele frequency spectrum in genome-wide human variation data reveals signals of differential demographic history in three large world populations. Genetics 166:351.

Riley R, Mathieson I, Mathieson S (2024) Interpreting generative adversarial networks to infer natural selection from genetic data. Genetics 226:iyae024.

Torada L, Lorenzon L, Beddis A, Isildak U, Pattini L, Mathieson S, Fumagalli M (2019) Imagene: a convolutional neural network to quantify natural selection from genomic data. BMC bioinformatics 20:337.

Van der Walt S, Schönberger JL, Nunez-Iglesias J, Boulogne F, Warner JD, Yager N, Gouillart E, Yu T (2014) scikit-image: image processing in python. PeerJ 2:e453.
